# 4-(4-Amino­phenyl­sulfon­yl)aniline–1,3,5-trinitro­benzene (1/2)

**DOI:** 10.1107/S1600536812001742

**Published:** 2012-01-21

**Authors:** Graham Smith, Urs D. Wermuth

**Affiliations:** a Faculty of Science and Technology, Queensland University of Technology, GPO Box 2434, Brisbane, Queensland 4001, Australia

## Abstract

The asymmetric unit of the title co-crystalline 1:2 adduct, C_12_H_12_N_2_O_2_·2C_6_H_3_N_3_O_6_, contains two independent mol­ecules of bis­(4-amino­phen­yl) sulfone (the drug Dapsone) and four mol­ecules of 1,3,5-trinitro­benzene and is extended into a two-dimensional hydrogen-bonded network structure through amino N—H⋯O hydrogen-bonding associations with nitro O-atom acceptors. In the two independent Dapsone mol­ecules, the inter-ring dihedral anges are 69.6 (3) and 63.63 (9)°. Aromatic π–π inter­actions are also found between one of the Dapsone aromatic rings and a trinitro­benzene ring [minimum ring centroid separation = 3.596 (3) Å]. A 4-amino­phenyl ring moiety of one of the Dapsone mol­ecules and two nitro groups of a trinitro­benzene are disordered in a 50:50 ratio.

## Related literature

For drug applications of Dapsone, see: Wilson *et al.* (1991 ▶). For the structures of Dapsone and its partial hydrate, see: Dickenson *et al.* (1970 ▶); Kus’mina *et al.* (1981 ▶). For the deca­rboxylation of 2,4,6-trinitro­benzoic acid and resultant co-crystal adduct structure, see: Smith *et al.* (2002 ▶).
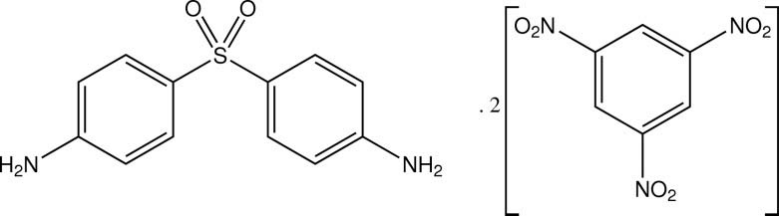


## Experimental

### 

#### Crystal data

C_12_H_12_N_2_O_2_S·2C_6_H_3_N_3_O_6_
*M* *_r_* = 674.52
Triclinic, 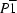

*a* = 8.3196 (2) Å
*b* = 18.3524 (5) Å
*c* = 18.6285 (5) Å
α = 83.577 (2)°
β = 87.539 (2)°
γ = 88.237 (2)°
*V* = 2822.86 (13) Å^3^
*Z* = 4
Mo *K*α radiation
μ = 0.20 mm^−1^
*T* = 200 K
0.35 × 0.15 × 0.10 mm

#### Data collection

Oxford Diffraction Gemini-S Ultra CCD detector diffractometer
Absorption correction: multi-scan (*CrysAlis PRO*; Oxford Diffraction, 2010 ▶) *T*_min_ = 0.932, *T*_max_ = 0.980
34593 measured reflections
11078 independent reflections
6613 reflections with *I* > 2σ(*I*)
*R*_int_ = 0.041

#### Refinement

*R*[*F* ^2^ > 2σ(*F* ^2^)] = 0.042
*wR*(*F* ^2^) = 0.077
*S* = 0.94
11078 reflections
880 parameters
H-atom parameters constrained
Δρ_max_ = 0.22 e Å^−3^
Δρ_min_ = −0.28 e Å^−3^

### 

Data collection: *CrysAlis PRO* (Oxford Diffraction, 2010 ▶); cell refinement: *CrysAlis PRO*; data reduction: *CrysAlis PRO*; program(s) used to solve structure: *SHELXS97* (Sheldrick, 2008 ▶); program(s) used to refine structure: *SHELXL97* (Sheldrick, 2008 ▶) within *WinGX* (Farrugia, 1999 ▶); molecular graphics: *PLATON* (Spek, 2009 ▶); software used to prepare material for publication: *PLATON*.

## Supplementary Material

Crystal structure: contains datablock(s) global, I. DOI: 10.1107/S1600536812001742/pv2497sup1.cif

Structure factors: contains datablock(s) I. DOI: 10.1107/S1600536812001742/pv2497Isup2.hkl

Supplementary material file. DOI: 10.1107/S1600536812001742/pv2497Isup3.cml

Additional supplementary materials:  crystallographic information; 3D view; checkCIF report

## Figures and Tables

**Table 1:** Hydrogen-bond geometry (Å, °)

*D*—H⋯*A*	*D*—H	H⋯*A*	*D*⋯*A*	*D*—H⋯*A*
N4*A*—H41*A*⋯O52*D*^i^	0.88	2.49	3.324 (4)	160
N41*A*—H43*A*⋯O12*D*^ii^	0.88	2.46	3.248 (2)	149
N41*A*—H44*A*⋯O52*C*^iii^	0.88	2.26	3.096 (2)	158
N4*X*—H4*X*1⋯O51*E*^iv^	0.88	2.46	3.267 (4)	152

Symmetry codes: (i) 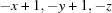; (ii) 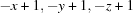; (iii) 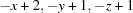; (iv) 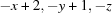.

**Fig. 1.**
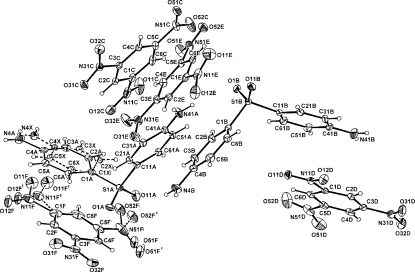
The molecular conformation and atom-numbering scheme for the two Dapsone molecules (A and B) and the four TNB molecules (C–F) in the asymmetric unit of the title co-crystal. The 50% disordered ring component [C1X–C6X, N4X] of the A ring is shown in dashed outline. Non-H atoms are shown as 30% probability displacement ellipsoids.

**Fig. 2.**
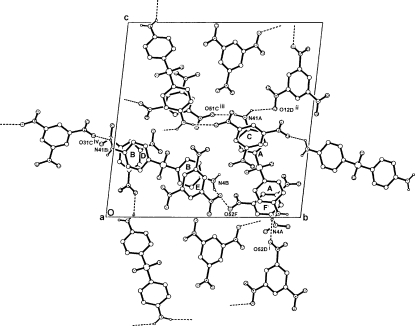
The hydrogen-bonding viewed down the a axial direction of the unit cell. The disordered ring component of the A Dapsone molecule is omitted together with non-interactive hydrogen atoms while hydrogen bonds are shown as dashed lines. Symmetry codes: i -x+1, -y+1, -z; ii -x+1, -y+1, -z+1; iii -x+2, -y+1, -z+1; iv x-1, y-1, z; v -x+2, -y+1, -z.

## supplementary crystallographic information

### Comment

Dapsone [4-(4-aminophenylsulfonyl)aniline] is a very weak Lewis base which finds use as an anti-leprotic, anti-malarial and leprostatic drug (Wilson *et al.*, 1991). The structure of the anhydrous parent compound is known (Dickenson *et al.*, 1970) and its partial (1/3) hydrate has been reported (Kus'mina *et al.*, 1981). However, no compounds or adducts of Dapsone are found in the crystallographic literature. Our attempted preparation of Dapsone with 2,4,6-trinitrobenzoic acid (TNBA) resulted in the title compound, the TNB adduct molecules resulting from the common facile decarboxylation of the parent acid (Smith *et al.*, 2002).

The asymmetric unit of the title adduct (Fig. 1) contains two independent molecules of Dapsone (*A* and *B*) and four molecules of 1,3,5-trinitrobenzene (TNB) (*C*–*F*) and is extended into a two-dimensional hydrogen-bonded network structure (Fig. 2) through amino N—H···O hydrogen-bond associations with nitro O-atom acceptors (Table 1). No interactions with sulfone O-atom acceptors are present. A weak π–π interaction is also found between one of the Dapsone aromatic ring moieties (C1*A*–C6*A*) and a TNB *F* ring [minimum ring centroid separation 3.719 (4) Å]. A 4-aminophenyl ring moiety of one of the Dapsone molecules (C1*A*–C6*A*, N4*A*) is 50% disordered, with the ring of the second component (C1*X*–C6*X*, N4*X*) giving a ring centroid separation of 3.596 (3) Å with the trinitrobenzene *A* ring. Two of the nitro groups of the *A* molecule are Also 50% disordered. In the two independent Dapsone molecules the inter-ring dihedral angles are 69.6 (3)° (*A*) and 63.63 (9)° (*B*) which compare with 77.3° in the anhydrous parent Dapsone molecule (Dickenson *et al.*, 1970) and 88.1, 75.8 and 74.7° for the three independent Dapsone molecules in the 0.33 hydrate structure (Kus'mina *et al.*, 1981).

### Experimental

The title compound was formed in an attempted synthesis of a proton-transfer salt of 4-(4-aminophenylsulfonyl)aniline (Dapsone) with 2,4,6-trinitrobenzoic acid by heating 1 mmol quantities of the two reagents in 50 ml of 50% ethanol–water for 15 min under reflux. Facile decarboxylation of the acid resulted in the formation of orange needle crystals of the title Dapsone- 1,3,5-trinitrobenzene co-crystal [m.p. 414–415 K], after room-temperature evaporation of the solvent.

### Refinement

All H atoms were included at calculated positions [N—H = 0.88 Å; C—H = 0.95 Å] and treated as riding, with *U*_iso_(H) = 1.2*U*_eq_(N/C). No reasonable acceptor atoms could be found for three of the H-atoms on separate amino groups (H42*A*, H42*B* and H44*B*).The atoms (C1–C6, N4) of Dapsone molecule *A* were disordered over two sites in a 50:50 ratio. Two nitro groups of the π-associated TNB *F* molecule (N11, O11, O12; N51, O51, O52) were also found to be similarly disordered in a 50:50 ratio.

### Crystal data

**Table:**

C_12_H_12_N_2_O_2_S·2C_6_H_3_N_3_O_6_	*Z* = 4
*M**_r_* = 674.52	*F*(000) = 1384
Triclinic, *P*1	*D*_x_ = 1.587 Mg m^−^^3^
Hall symbol: -P 1	Melting point > 414 K
*a* = 8.3196 (2) Å	Mo *K*α radiation, λ = 0.71073 Å
*b* = 18.3524 (5) Å	Cell parameters from 10417 reflections
*c* = 18.6285 (5) Å	θ = 3.2–28.8°
α = 83.577 (2)°	µ = 0.20 mm^−^^1^
β = 87.539 (2)°	*T* = 200 K
γ = 88.237 (2)°	Needle, orange
*V* = 2822.86 (13) Å^3^	0.35 × 0.15 × 0.10 mm

### Data collection

**Table:**

Oxford Diffraction Gemini-S Ultra CCD detector diffractometer	11078 independent reflections
Radiation source: Enhance (Mo) X-ray source	6613 reflections with *I* > 2σ(*I*)
graphite	*R*_int_ = 0.041
Detector resolution: 16.077 pixels mm^-1^	θ_max_ = 26.0°, θ_min_ = 3.2°
ω scans	*h* = −10→10
Absorption correction: multi-scan (CrysAlis PRO; Oxford Diffraction, 2010)	*k* = −22→22
*T*_min_ = 0.932, *T*_max_ = 0.980	*l* = −22→22
34593 measured reflections	

### Refinement

**Table:**

Refinement on *F*^2^	Primary atom site location: structure-invariant direct methods
Least-squares matrix: full	Secondary atom site location: difference Fourier map
*R*[*F*^2^ > 2σ(*F*^2^)] = 0.042	Hydrogen site location: inferred from neighbouring sites
*wR*(*F*^2^) = 0.077	H-atom parameters constrained
*S* = 0.94	*w* = 1/[σ^2^(*F*_o_^2^) + (0.0308*P*)^2^] where *P* = (*F*_o_^2^ + 2*F*_c_^2^)/3
11078 reflections	(Δ/σ)_max_ < 0.001
880 parameters	Δρ_max_ = 0.22 e Å^−^^3^
0 restraints	Δρ_min_ = −0.28 e Å^−^^3^

### Special details

**Table:**

Geometry. Bond distances, angles *etc*. have been calculated using the rounded fractional coordinates. All su's are estimated from the variances of the (full) variance-covariance matrix. The cell e.s.d.'s are taken into account in the estimation of distances, angles and torsion angles

### Fractional atomic coordinates and isotropic or equivalent isotropic displacement parameters (Å^2^)

**Table:**

	*x*	*y*	*z*	*U*_iso_*/*U*_eq_	Occ. (<1)
S1A	0.38876 (6)	0.76073 (3)	0.25467 (3)	0.0376 (2)	
O1A	0.28914 (15)	0.82213 (8)	0.27305 (7)	0.0514 (5)	
O11A	0.31123 (19)	0.69889 (8)	0.23122 (9)	0.0671 (6)	
N4A	0.8310 (4)	0.9143 (2)	0.0302 (2)	0.0590 (10)	0.500
N4X	0.8262 (4)	0.8502 (2)	0.0096 (2)	0.0590 (10)	0.500
N41A	0.7501 (2)	0.65642 (9)	0.51329 (9)	0.0509 (7)	
C1A	0.5240 (2)	0.79555 (12)	0.18603 (10)	0.0346 (7)	
C2A	0.6061 (12)	0.7655 (3)	0.1359 (6)	0.035 (2)	0.500
C2X	0.5755 (12)	0.7366 (3)	0.1391 (6)	0.035 (2)	0.500
C3A	0.7078 (9)	0.8002 (4)	0.0815 (4)	0.0466 (18)	0.500
C3X	0.6748 (7)	0.7578 (3)	0.0837 (4)	0.0466 (18)	0.500
C4A	0.7298 (6)	0.8744 (3)	0.0809 (3)	0.0326 (16)	0.500
C4X	0.7245 (7)	0.8276 (4)	0.0681 (3)	0.0326 (16)	0.500
C5A	0.6510 (7)	0.9137 (3)	0.1356 (3)	0.0348 (16)	0.500
C5X	0.6747 (7)	0.8817 (3)	0.1140 (3)	0.0348 (16)	0.500
C6A	0.5532 (17)	0.8792 (6)	0.1865 (8)	0.029 (3)	0.500
C6X	0.5696 (16)	0.8578 (6)	0.1744 (7)	0.029 (3)	0.500
C11A	0.4985 (2)	0.72898 (10)	0.33046 (10)	0.0305 (7)	
C21A	0.5435 (2)	0.77863 (11)	0.37641 (11)	0.0340 (7)	
C31A	0.6271 (2)	0.75444 (11)	0.43705 (10)	0.0353 (7)	
C41A	0.6673 (2)	0.68028 (10)	0.45335 (10)	0.0327 (7)	
C51A	0.6221 (2)	0.63100 (10)	0.40587 (10)	0.0340 (7)	
C61A	0.5395 (2)	0.65541 (11)	0.34504 (10)	0.0337 (7)	
S1B	0.68365 (6)	0.26463 (3)	0.26762 (3)	0.0373 (2)	
O1B	0.77419 (15)	0.25066 (7)	0.20232 (8)	0.0500 (5)	
O11B	0.77013 (16)	0.27879 (7)	0.32980 (8)	0.0538 (6)	
N4B	0.2836 (2)	0.53029 (11)	0.18608 (15)	0.0924 (12)	
N41B	0.3165 (2)	−0.00479 (10)	0.35065 (13)	0.0722 (8)	
C1B	0.5569 (2)	0.34105 (10)	0.24448 (11)	0.0344 (7)	
C2B	0.5190 (2)	0.36012 (11)	0.17346 (12)	0.0434 (8)	
C3B	0.4285 (3)	0.42275 (13)	0.15398 (14)	0.0565 (9)	
C4B	0.3753 (3)	0.46766 (12)	0.20613 (18)	0.0602 (9)	
C5B	0.4125 (3)	0.44756 (13)	0.27736 (16)	0.0593 (10)	
C6B	0.5017 (2)	0.38509 (12)	0.29676 (13)	0.0467 (8)	
C11B	0.5658 (2)	0.18755 (10)	0.29337 (11)	0.0302 (7)	
C21B	0.5071 (2)	0.14899 (11)	0.24066 (11)	0.0397 (8)	
C31B	0.4241 (2)	0.08572 (12)	0.25962 (13)	0.0464 (9)	
C41B	0.3983 (2)	0.05920 (11)	0.33157 (14)	0.0449 (8)	
C51B	0.4557 (2)	0.09851 (11)	0.38467 (12)	0.0440 (8)	
C61B	0.5384 (2)	0.16223 (11)	0.36521 (11)	0.0371 (7)	
O11'	0.5149 (8)	0.8058 (3)	−0.0726 (3)	0.0832 (19)	0.500
O11F	0.5379 (9)	0.8540 (3)	−0.0733 (3)	0.0832 (19)	0.500
O12'	0.4743 (9)	0.9220 (3)	−0.0530 (4)	0.0775 (18)	0.500
O12F	0.4741 (8)	0.9631 (3)	−0.0398 (3)	0.0775 (18)	0.500
O31F	0.1593 (2)	0.97159 (10)	0.17118 (12)	0.0933 (9)	
O32F	−0.0153 (2)	0.89062 (9)	0.20991 (9)	0.0627 (7)	
O51'	−0.0255 (11)	0.6561 (7)	0.1189 (6)	0.064 (3)	0.500
O51F	0.0374 (11)	0.6585 (6)	0.1008 (6)	0.064 (3)	0.500
O52'	0.1862 (9)	0.6229 (4)	0.0546 (4)	0.073 (3)	0.500
O52F	0.2438 (9)	0.6459 (4)	0.0297 (4)	0.073 (3)	0.500
N11'	0.4541 (13)	0.8584 (4)	−0.0442 (5)	0.058 (3)	0.500
N11F	0.4552 (13)	0.8952 (4)	−0.0374 (5)	0.058 (3)	0.500
N31F	0.0945 (2)	0.91286 (11)	0.16936 (12)	0.0558 (8)	
N51F	0.1273 (4)	0.67557 (15)	0.07376 (17)	0.0821 (14)	
C1F	0.3291 (3)	0.8485 (2)	0.01513 (13)	0.0704 (12)	
C2F	0.2697 (3)	0.89427 (14)	0.06317 (14)	0.0540 (9)	
C3F	0.1566 (2)	0.86603 (12)	0.11448 (12)	0.0397 (8)	
C4F	0.1026 (2)	0.79570 (11)	0.11831 (11)	0.0400 (8)	
C5F	0.1713 (3)	0.75304 (13)	0.06926 (13)	0.0525 (9)	
C6F	0.2837 (4)	0.7777 (2)	0.01717 (14)	0.0722 (13)	
O11C	0.9598 (3)	0.61193 (11)	0.26861 (10)	0.1009 (9)	
O12C	0.8668 (2)	0.72426 (11)	0.26112 (9)	0.0712 (7)	
O31C	1.07076 (18)	0.88686 (8)	0.41772 (8)	0.0551 (6)	
O32C	1.25965 (19)	0.84718 (8)	0.48865 (9)	0.0547 (6)	
O51C	1.36284 (19)	0.58561 (9)	0.53825 (9)	0.0592 (6)	
O52C	1.2528 (3)	0.51322 (9)	0.47361 (10)	0.0963 (9)	
N11C	1.1543 (2)	0.83754 (9)	0.44757 (10)	0.0392 (6)	
N31C	0.9431 (3)	0.67244 (14)	0.29009 (11)	0.0614 (9)	
N51C	1.2733 (3)	0.57343 (11)	0.49189 (11)	0.0518 (8)	
C1C	1.0269 (2)	0.68293 (12)	0.35632 (11)	0.0395 (8)	
C2C	1.0372 (2)	0.75278 (11)	0.37383 (11)	0.0353 (7)	
C3C	1.1278 (2)	0.76201 (10)	0.43196 (10)	0.0301 (7)	
C4C	1.2042 (2)	0.70505 (10)	0.47255 (10)	0.0328 (7)	
C5C	1.1870 (2)	0.63621 (11)	0.45280 (11)	0.0359 (7)	
C6C	1.0989 (3)	0.62355 (12)	0.39452 (11)	0.0438 (8)	
O11D	0.18351 (17)	0.24154 (8)	0.33582 (8)	0.0468 (5)	
O12D	0.10205 (17)	0.19402 (8)	0.44197 (8)	0.0525 (6)	
O31D	−0.2163 (2)	−0.02162 (9)	0.46941 (12)	0.0719 (7)	
O32D	−0.2969 (2)	−0.06595 (10)	0.37460 (12)	0.0894 (9)	
O51D	−0.1722 (2)	0.06064 (11)	0.13729 (10)	0.0937 (9)	
O52D	0.0215 (3)	0.13759 (12)	0.12532 (10)	0.0942 (9)	
N11D	0.11084 (19)	0.19562 (9)	0.37591 (10)	0.0356 (6)	
N31D	−0.2226 (2)	−0.02208 (10)	0.40421 (15)	0.0568 (8)	
N51D	−0.0715 (3)	0.09712 (12)	0.16134 (12)	0.0623 (9)	
C1D	0.0261 (2)	0.13858 (10)	0.34367 (10)	0.0278 (6)	
C2D	−0.0500 (2)	0.08493 (10)	0.38886 (11)	0.0320 (7)	
C3D	−0.1353 (2)	0.03493 (10)	0.35704 (12)	0.0364 (7)	
C4D	−0.1457 (2)	0.03741 (11)	0.28337 (13)	0.0434 (8)	
C5D	−0.0634 (2)	0.09174 (11)	0.24105 (11)	0.0381 (8)	
C6D	0.0245 (2)	0.14302 (10)	0.26951 (11)	0.0331 (7)	
O11E	1.0370 (2)	0.40646 (12)	0.35729 (9)	0.0855 (9)	
O12E	0.8341 (2)	0.48221 (10)	0.34813 (9)	0.0778 (7)	
O31E	0.6554 (2)	0.57439 (9)	0.11041 (10)	0.0728 (7)	
O32E	0.7634 (2)	0.52991 (10)	0.01659 (11)	0.0872 (9)	
O51E	1.0834 (2)	0.30715 (9)	0.05044 (9)	0.0807 (8)	
O52E	1.19387 (19)	0.27635 (8)	0.15271 (9)	0.0590 (6)	
N11E	0.9344 (3)	0.44178 (12)	0.32300 (11)	0.0538 (8)	
N31E	0.7460 (2)	0.53190 (10)	0.08200 (13)	0.0551 (8)	
N51E	1.1046 (2)	0.31519 (10)	0.11382 (11)	0.0497 (8)	
C1E	0.9312 (2)	0.43540 (11)	0.24451 (11)	0.0363 (8)	
C2E	0.8385 (2)	0.48504 (11)	0.20222 (12)	0.0398 (8)	
C3E	0.8420 (2)	0.47900 (11)	0.12899 (12)	0.0382 (7)	
C4E	0.9293 (2)	0.42476 (11)	0.09874 (11)	0.0397 (8)	
C5E	1.0170 (2)	0.37599 (10)	0.14410 (11)	0.0352 (7)	
C6E	1.0227 (2)	0.38071 (11)	0.21716 (11)	0.0366 (7)	
H4X1	0.86200	0.81850	−0.01990	0.0710*	0.500
H4X2	0.85490	0.89620	0.00180	0.0710*	0.500
H2A	0.59610	0.71410	0.13600	0.0420*	0.500
H2X	0.53930	0.68770	0.14850	0.0420*	0.500
H3A	0.76010	0.77340	0.04610	0.0560*	0.500
H3X	0.71310	0.72220	0.05330	0.0560*	0.500
H5A	0.66840	0.96470	0.13550	0.0420*	0.500
H5X	0.70910	0.93090	0.10510	0.0420*	0.500
H6A	0.50200	0.90540	0.22270	0.0350*	0.500
H6X	0.53430	0.89220	0.20650	0.0350*	0.500
H21A	0.51670	0.82930	0.36600	0.0410*	
H31A	0.65780	0.78870	0.46820	0.0420*	
H41A	0.88420	0.89210	−0.00330	0.0710*	0.500
H42A	0.84150	0.96160	0.03180	0.0710*	0.500
H43A	0.77900	0.68790	0.54230	0.0610*	
H44A	0.77480	0.60950	0.52300	0.0610*	
H51A	0.64890	0.58030	0.41580	0.0410*	
H61A	0.51060	0.62170	0.31300	0.0400*	
H2B	0.55560	0.32980	0.13760	0.0520*	
H3B	0.40230	0.43530	0.10490	0.0680*	
H5B	0.37550	0.47750	0.31340	0.0710*	
H6B	0.52580	0.37190	0.34600	0.0560*	
H21B	0.52440	0.16650	0.19110	0.0480*	
H31B	0.38410	0.05990	0.22300	0.0560*	
H41B	0.24760	0.55830	0.21900	0.1110*	
H42B	0.26130	0.54210	0.14040	0.1110*	
H43B	0.28020	−0.02920	0.31690	0.0870*	
H44B	0.30070	−0.02120	0.39660	0.0870*	
H51B	0.43790	0.08140	0.43430	0.0530*	
H61B	0.57670	0.18880	0.40160	0.0450*	
H2F	0.30440	0.94330	0.06150	0.0650*	
H4F	0.02220	0.77780	0.15300	0.0480*	
H6F	0.32850	0.74650	−0.01630	0.0860*	
H2C	0.98430	0.79300	0.34720	0.0420*	
H4C	1.26600	0.71310	0.51250	0.0390*	
H6C	1.08860	0.57540	0.38140	0.0530*	
H2D	−0.04410	0.08240	0.43990	0.0380*	
H4D	−0.20710	0.00310	0.26240	0.0520*	
H6D	0.08130	0.17970	0.23950	0.0400*	
H2E	0.77470	0.52190	0.22260	0.0480*	
H4E	0.92920	0.42100	0.04830	0.0480*	
H6E	1.08720	0.34760	0.24730	0.0440*	

### Atomic displacement parameters (Å^2^)

**Table:**

	*U*^11^	*U*^22^	*U*^33^	*U*^12^	*U*^13^	*U*^23^
S1A	0.0317 (3)	0.0441 (3)	0.0353 (3)	−0.0037 (3)	−0.0080 (2)	0.0057 (3)
O1A	0.0360 (8)	0.0693 (11)	0.0431 (9)	0.0214 (8)	0.0034 (7)	0.0111 (8)
O11A	0.0771 (11)	0.0574 (11)	0.0677 (11)	−0.0303 (9)	−0.0429 (9)	0.0119 (9)
N4A	0.0402 (14)	0.088 (2)	0.0441 (18)	−0.0020 (19)	0.0090 (14)	0.0093 (18)
N4X	0.0402 (14)	0.088 (2)	0.0441 (18)	−0.0020 (19)	0.0090 (14)	0.0093 (18)
N41A	0.0749 (13)	0.0345 (11)	0.0443 (12)	−0.0022 (9)	−0.0296 (10)	0.0012 (9)
C1A	0.0286 (11)	0.0487 (14)	0.0251 (12)	0.0100 (10)	−0.0058 (9)	0.0012 (11)
C2A	0.042 (4)	0.026 (4)	0.0388 (18)	0.008 (3)	−0.013 (2)	−0.010 (4)
C2X	0.042 (4)	0.026 (4)	0.0388 (18)	0.008 (3)	−0.013 (2)	−0.010 (4)
C3A	0.045 (3)	0.055 (4)	0.036 (2)	0.013 (3)	0.0011 (19)	0.007 (4)
C3X	0.045 (3)	0.055 (4)	0.036 (2)	0.013 (3)	0.0011 (19)	0.007 (4)
C4A	0.0231 (16)	0.057 (4)	0.017 (2)	0.009 (3)	−0.0010 (14)	−0.004 (3)
C4X	0.0231 (16)	0.057 (4)	0.017 (2)	0.009 (3)	−0.0010 (14)	−0.004 (3)
C5A	0.032 (2)	0.038 (3)	0.034 (3)	0.000 (3)	−0.005 (2)	−0.001 (2)
C5X	0.032 (2)	0.038 (3)	0.034 (3)	0.000 (3)	−0.005 (2)	−0.001 (2)
C6A	0.031 (3)	0.021 (6)	0.037 (4)	0.005 (3)	−0.004 (2)	−0.012 (3)
C6X	0.031 (3)	0.021 (6)	0.037 (4)	0.005 (3)	−0.004 (2)	−0.012 (3)
C11A	0.0262 (10)	0.0339 (12)	0.0305 (12)	−0.0049 (9)	−0.0031 (9)	0.0021 (9)
C21A	0.0361 (11)	0.0264 (11)	0.0385 (13)	−0.0001 (9)	−0.0026 (10)	0.0006 (10)
C31A	0.0408 (12)	0.0307 (12)	0.0358 (13)	−0.0062 (9)	−0.0079 (10)	−0.0066 (10)
C41A	0.0359 (11)	0.0314 (12)	0.0301 (12)	−0.0039 (9)	−0.0061 (9)	0.0017 (10)
C51A	0.0421 (12)	0.0236 (11)	0.0355 (12)	−0.0016 (9)	−0.0046 (10)	0.0011 (9)
C61A	0.0403 (12)	0.0307 (12)	0.0309 (12)	−0.0067 (9)	−0.0032 (10)	−0.0042 (9)
S1B	0.0287 (3)	0.0283 (3)	0.0534 (4)	−0.0025 (2)	−0.0064 (3)	0.0047 (3)
O1B	0.0386 (8)	0.0394 (9)	0.0673 (11)	0.0012 (7)	0.0173 (8)	0.0064 (8)
O11B	0.0470 (9)	0.0418 (9)	0.0735 (11)	−0.0099 (7)	−0.0330 (8)	0.0039 (8)
N4B	0.0573 (13)	0.0454 (13)	0.167 (3)	0.0159 (11)	−0.0117 (15)	0.0187 (15)
N41B	0.0598 (13)	0.0407 (12)	0.1129 (18)	−0.0171 (10)	0.0085 (13)	0.0056 (12)
C1B	0.0284 (11)	0.0272 (11)	0.0472 (14)	−0.0048 (9)	−0.0046 (10)	0.0004 (10)
C2B	0.0406 (13)	0.0365 (13)	0.0530 (15)	−0.0021 (10)	−0.0102 (11)	−0.0014 (11)
C3B	0.0477 (14)	0.0428 (15)	0.0765 (19)	−0.0083 (12)	−0.0264 (13)	0.0156 (14)
C4B	0.0311 (13)	0.0288 (14)	0.117 (2)	−0.0011 (10)	−0.0060 (15)	0.0090 (16)
C5B	0.0483 (15)	0.0386 (15)	0.091 (2)	0.0009 (12)	0.0087 (15)	−0.0125 (15)
C6B	0.0446 (13)	0.0387 (13)	0.0566 (15)	−0.0033 (11)	0.0002 (12)	−0.0045 (12)
C11B	0.0266 (10)	0.0258 (11)	0.0372 (13)	−0.0007 (8)	−0.0027 (9)	0.0015 (9)
C21B	0.0397 (12)	0.0441 (14)	0.0351 (13)	−0.0032 (10)	−0.0015 (10)	−0.0032 (11)
C31B	0.0389 (13)	0.0416 (14)	0.0614 (17)	−0.0077 (11)	−0.0060 (12)	−0.0145 (12)
C41B	0.0285 (12)	0.0282 (13)	0.0757 (18)	−0.0014 (9)	0.0062 (12)	0.0017 (12)
C51B	0.0425 (13)	0.0409 (14)	0.0431 (14)	0.0044 (11)	0.0070 (11)	0.0143 (11)
C61B	0.0352 (12)	0.0359 (12)	0.0400 (14)	0.0028 (10)	−0.0054 (10)	−0.0029 (10)
O11'	0.092 (2)	0.117 (5)	0.0410 (14)	−0.002 (4)	0.0015 (14)	−0.012 (3)
O11F	0.092 (2)	0.117 (5)	0.0410 (14)	−0.002 (4)	0.0015 (14)	−0.012 (3)
O12'	0.0806 (17)	0.071 (4)	0.077 (3)	−0.038 (4)	−0.0293 (18)	0.029 (3)
O12F	0.0806 (17)	0.071 (4)	0.077 (3)	−0.038 (4)	−0.0293 (18)	0.029 (3)
O31F	0.0742 (13)	0.0491 (12)	0.166 (2)	−0.0032 (10)	−0.0150 (13)	−0.0486 (13)
O32F	0.0468 (10)	0.0728 (13)	0.0723 (13)	0.0154 (9)	−0.0068 (9)	−0.0275 (10)
O51'	0.087 (7)	0.0425 (14)	0.063 (5)	−0.020 (4)	0.001 (4)	−0.001 (3)
O51F	0.087 (7)	0.0425 (14)	0.063 (5)	−0.020 (4)	0.001 (4)	−0.001 (3)
O52'	0.100 (5)	0.037 (3)	0.086 (5)	0.014 (2)	−0.023 (3)	−0.025 (3)
O52F	0.100 (5)	0.037 (3)	0.086 (5)	0.014 (2)	−0.023 (3)	−0.025 (3)
N11'	0.061 (2)	0.076 (7)	0.039 (2)	0.016 (5)	−0.0221 (17)	−0.010 (5)
N11F	0.061 (2)	0.076 (7)	0.039 (2)	0.016 (5)	−0.0221 (17)	−0.010 (5)
N31F	0.0421 (12)	0.0469 (14)	0.0826 (17)	0.0120 (11)	−0.0209 (12)	−0.0223 (12)
N51F	0.122 (3)	0.054 (2)	0.078 (2)	0.0342 (18)	−0.0631 (19)	−0.0293 (16)
C1F	0.0444 (16)	0.136 (3)	0.0282 (15)	0.0172 (19)	−0.0141 (13)	0.0013 (18)
C2F	0.0412 (14)	0.0636 (17)	0.0546 (17)	−0.0026 (12)	−0.0251 (13)	0.0140 (14)
C3F	0.0346 (12)	0.0406 (14)	0.0462 (14)	0.0096 (10)	−0.0202 (11)	−0.0107 (12)
C4F	0.0399 (12)	0.0386 (13)	0.0430 (14)	0.0068 (10)	−0.0203 (11)	−0.0066 (11)
C5F	0.0685 (17)	0.0486 (16)	0.0443 (16)	0.0230 (13)	−0.0310 (14)	−0.0176 (13)
C6F	0.072 (2)	0.111 (3)	0.0376 (17)	0.0429 (19)	−0.0265 (15)	−0.0292 (18)
O11C	0.178 (2)	0.0708 (14)	0.0599 (13)	−0.0583 (14)	−0.0368 (13)	−0.0059 (11)
O12C	0.0562 (11)	0.1047 (16)	0.0502 (11)	−0.0182 (10)	−0.0188 (9)	0.0135 (11)
O31C	0.0645 (10)	0.0328 (9)	0.0648 (11)	0.0111 (8)	0.0039 (9)	0.0032 (8)
O32C	0.0604 (10)	0.0449 (10)	0.0633 (11)	−0.0057 (8)	−0.0109 (9)	−0.0212 (8)
O51C	0.0649 (11)	0.0585 (11)	0.0499 (11)	0.0078 (9)	−0.0064 (9)	0.0120 (9)
O52C	0.173 (2)	0.0268 (10)	0.0910 (15)	0.0110 (11)	−0.0330 (14)	−0.0085 (10)
N11C	0.0445 (11)	0.0305 (11)	0.0418 (11)	0.0008 (9)	0.0080 (9)	−0.0044 (9)
N31C	0.0719 (15)	0.0686 (16)	0.0441 (14)	−0.0408 (13)	−0.0111 (11)	0.0057 (12)
N51C	0.0752 (15)	0.0358 (13)	0.0414 (12)	0.0049 (11)	0.0045 (11)	0.0050 (10)
C1C	0.0437 (13)	0.0448 (14)	0.0305 (12)	−0.0210 (10)	−0.0024 (10)	−0.0001 (11)
C2C	0.0304 (11)	0.0382 (13)	0.0354 (13)	−0.0054 (9)	0.0026 (10)	0.0041 (10)
C3C	0.0300 (11)	0.0276 (11)	0.0322 (12)	−0.0015 (9)	0.0050 (9)	−0.0033 (9)
C4C	0.0343 (11)	0.0351 (12)	0.0294 (12)	−0.0054 (9)	0.0021 (9)	−0.0048 (10)
C5C	0.0479 (13)	0.0273 (12)	0.0310 (12)	−0.0028 (10)	0.0038 (10)	0.0019 (10)
C6C	0.0640 (15)	0.0316 (13)	0.0361 (13)	−0.0164 (11)	0.0023 (12)	−0.0028 (10)
O11D	0.0498 (9)	0.0346 (9)	0.0554 (10)	−0.0139 (7)	−0.0074 (8)	0.0036 (8)
O12D	0.0627 (10)	0.0622 (11)	0.0361 (10)	−0.0180 (8)	−0.0029 (8)	−0.0162 (8)
O31D	0.0836 (13)	0.0421 (10)	0.0847 (14)	−0.0051 (9)	0.0331 (12)	0.0034 (10)
O32D	0.0762 (13)	0.0443 (11)	0.1481 (19)	−0.0303 (10)	−0.0290 (13)	0.0046 (12)
O51D	0.1170 (16)	0.0956 (15)	0.0795 (14)	0.0097 (13)	−0.0617 (13)	−0.0396 (12)
O52D	0.167 (2)	0.0766 (15)	0.0412 (12)	−0.0144 (14)	−0.0097 (13)	−0.0116 (11)
N11D	0.0350 (10)	0.0310 (10)	0.0419 (12)	−0.0001 (8)	−0.0061 (9)	−0.0076 (9)
N31D	0.0420 (12)	0.0291 (12)	0.0970 (19)	0.0020 (9)	0.0048 (13)	−0.0001 (13)
N51D	0.0900 (18)	0.0486 (14)	0.0531 (16)	0.0211 (13)	−0.0313 (14)	−0.0218 (12)
C1D	0.0257 (10)	0.0242 (11)	0.0346 (12)	0.0019 (8)	−0.0034 (9)	−0.0083 (9)
C2D	0.0333 (11)	0.0260 (11)	0.0367 (12)	0.0059 (9)	−0.0010 (10)	−0.0060 (10)
C3D	0.0293 (11)	0.0216 (11)	0.0589 (16)	0.0030 (9)	−0.0002 (11)	−0.0083 (10)
C4D	0.0388 (12)	0.0270 (12)	0.0692 (18)	0.0083 (10)	−0.0229 (12)	−0.0210 (12)
C5D	0.0473 (13)	0.0307 (12)	0.0391 (14)	0.0115 (10)	−0.0170 (11)	−0.0137 (11)
C6D	0.0336 (11)	0.0271 (11)	0.0385 (13)	0.0082 (9)	−0.0045 (10)	−0.0051 (10)
O11E	0.0944 (15)	0.1181 (17)	0.0475 (12)	0.0032 (13)	−0.0249 (11)	−0.0178 (11)
O12E	0.1133 (15)	0.0687 (12)	0.0515 (11)	−0.0099 (11)	0.0321 (11)	−0.0192 (10)
O31E	0.0678 (12)	0.0477 (11)	0.1024 (15)	0.0130 (9)	−0.0069 (11)	−0.0086 (10)
O32E	0.1282 (17)	0.0727 (14)	0.0597 (13)	0.0223 (12)	−0.0317 (12)	0.0008 (11)
O51E	0.1281 (16)	0.0698 (13)	0.0460 (11)	0.0309 (11)	−0.0065 (11)	−0.0224 (10)
O52E	0.0651 (11)	0.0416 (10)	0.0708 (12)	0.0081 (8)	−0.0112 (9)	−0.0083 (9)
N11E	0.0673 (15)	0.0548 (14)	0.0414 (13)	−0.0252 (11)	0.0085 (12)	−0.0122 (11)
N31E	0.0619 (14)	0.0349 (12)	0.0694 (16)	−0.0041 (10)	−0.0147 (12)	−0.0045 (12)
N51E	0.0656 (14)	0.0366 (12)	0.0473 (14)	0.0010 (10)	−0.0013 (11)	−0.0075 (10)
C1E	0.0423 (13)	0.0357 (13)	0.0327 (13)	−0.0170 (10)	0.0026 (10)	−0.0085 (10)
C2E	0.0357 (12)	0.0342 (13)	0.0519 (15)	−0.0119 (10)	0.0052 (11)	−0.0152 (11)
C3E	0.0413 (12)	0.0288 (12)	0.0456 (14)	−0.0059 (10)	−0.0070 (11)	−0.0053 (11)
C4E	0.0534 (14)	0.0349 (13)	0.0321 (12)	−0.0082 (11)	−0.0017 (11)	−0.0073 (10)
C5E	0.0423 (12)	0.0254 (11)	0.0387 (13)	−0.0059 (9)	−0.0002 (10)	−0.0061 (10)
C6E	0.0387 (12)	0.0350 (12)	0.0361 (13)	−0.0146 (10)	−0.0038 (10)	0.0005 (10)

### Geometric parameters (Å, °)

**Table:**

S1A—O1A	1.4417 (15)	N11E—C1E	1.481 (3)
S1A—O11A	1.4405 (16)	C21A—C31A	1.376 (3)
S1A—C1A	1.7456 (19)	C31A—C41A	1.395 (3)
S1A—C11A	1.7494 (19)	N31E—C3E	1.473 (3)
S1B—C1B	1.7543 (19)	C41A—C51A	1.404 (3)
S1B—O1B	1.4464 (15)	C51A—C61A	1.375 (3)
S1B—O11B	1.4413 (15)	N51E—C5E	1.466 (3)
S1B—C11B	1.7566 (19)	C2A—H2A	0.9500
O11'—N11'	1.237 (10)	C2X—H2X	0.9500
O11F—N11F	1.239 (11)	C3A—H3A	0.9500
O12'—N11'	1.177 (9)	C3X—H3X	0.9500
O12F—N11F	1.256 (9)	C5A—H5A	0.9500
O31F—N31F	1.224 (3)	C5X—H5X	0.9500
O32F—N31F	1.211 (3)	C6A—H6A	0.9500
O51'—N51F	1.523 (10)	C6X—H6X	0.9500
O51F—N51F	0.926 (10)	C21A—H21A	0.9500
O52'—N51F	1.155 (8)	C31A—H31A	0.9500
O52F—N51F	1.382 (8)	C51A—H51A	0.9500
O11C—N31C	1.224 (3)	C61A—H61A	0.9500
O12C—N31C	1.215 (3)	C1B—C6B	1.388 (3)
O31C—N11C	1.222 (2)	C1B—C2B	1.378 (3)
O32C—N11C	1.218 (2)	C2B—C3B	1.377 (3)
O51C—N51C	1.208 (3)	C3B—C4B	1.392 (4)
O52C—N51C	1.211 (3)	C4B—C5B	1.383 (4)
O11D—N11D	1.219 (2)	C5B—C6B	1.369 (3)
O12D—N11D	1.227 (2)	C11B—C61B	1.378 (3)
O31D—N31D	1.219 (4)	C11B—C21B	1.386 (3)
O32D—N31D	1.222 (3)	C21B—C31B	1.374 (3)
O51D—N51D	1.219 (3)	C31B—C41B	1.384 (3)
O52D—N51D	1.217 (3)	C41B—C51B	1.396 (3)
N4A—C4A	1.397 (6)	C51B—C61B	1.380 (3)
N4X—C4X	1.385 (7)	C2B—H2B	0.9500
O11E—N11E	1.215 (3)	C3B—H3B	0.9500
O12E—N11E	1.213 (3)	C5B—H5B	0.9500
O31E—N31E	1.216 (3)	C6B—H6B	0.9500
O32E—N31E	1.225 (3)	C21B—H21B	0.9500
N41A—C41A	1.361 (2)	C31B—H31B	0.9500
O51E—N51E	1.227 (3)	C51B—H51B	0.9500
O52E—N51E	1.218 (2)	C61B—H61B	0.9500
N4A—H42A	0.8800	C1F—C2F	1.362 (4)
N4A—H41A	0.8800	C1F—C6F	1.360 (5)
N4X—H4X2	0.8800	C2F—C3F	1.382 (3)
N4X—H4X1	0.8800	C3F—C4F	1.373 (3)
N41A—H43A	0.8800	C4F—C5F	1.365 (3)
N41A—H44A	0.8800	C5F—C6F	1.368 (4)
N4B—C4B	1.385 (3)	C2F—H2F	0.9500
N41B—C41B	1.380 (3)	C4F—H4F	0.9500
N4B—H42B	0.8800	C6F—H6F	0.9500
N4B—H41B	0.8800	C1C—C6C	1.370 (3)
N41B—H44B	0.8800	C1C—C2C	1.364 (3)
N41B—H43B	0.8800	C2C—C3C	1.374 (3)
N11'—C1F	1.485 (10)	C3C—C4C	1.376 (3)
N11F—C1F	1.604 (10)	C4C—C5C	1.368 (3)
N31F—C3F	1.475 (3)	C5C—C6C	1.380 (3)
N51F—C5F	1.471 (4)	C2C—H2C	0.9500
N11C—C3C	1.473 (2)	C4C—H4C	0.9500
N31C—C1C	1.477 (3)	C6C—H6C	0.9500
N51C—C5C	1.476 (3)	C1D—C2D	1.373 (3)
N11D—C1D	1.475 (2)	C1D—C6D	1.375 (3)
N31D—C3D	1.477 (3)	C2D—C3D	1.377 (3)
N51D—C5D	1.481 (3)	C3D—C4D	1.374 (3)
C1A—C6A	1.563 (11)	C4D—C5D	1.380 (3)
C1A—C2X	1.506 (8)	C5D—C6D	1.374 (3)
C1A—C2A	1.296 (10)	C2D—H2D	0.9500
C1A—C6X	1.208 (12)	C4D—H4D	0.9500
C2A—C3A	1.401 (13)	C6D—H6D	0.9500
C2X—C3X	1.325 (13)	C1E—C2E	1.376 (3)
C3A—C4A	1.378 (9)	C1E—C6E	1.371 (3)
C3X—C4X	1.355 (9)	C2E—C3E	1.380 (3)
C4A—C5A	1.438 (8)	C3E—C4E	1.372 (3)
C4X—C5X	1.422 (9)	C4E—C5E	1.375 (3)
C5A—C6A	1.339 (15)	C5E—C6E	1.376 (3)
C5X—C6X	1.432 (14)	C2E—H2E	0.9500
C11A—C21A	1.388 (3)	C4E—H4E	0.9500
C11A—C61A	1.382 (3)	C6E—H6E	0.9500
			
O1A—S1A—O11A	118.15 (9)	C21A—C31A—H31A	120.00
O1A—S1A—C1A	106.20 (9)	C41A—C31A—H31A	120.00
O1A—S1A—C11A	107.65 (9)	C41A—C51A—H51A	120.00
O11A—S1A—C1A	108.53 (10)	C61A—C51A—H51A	120.00
O11A—S1A—C11A	107.83 (9)	C11A—C61A—H61A	120.00
C1A—S1A—C11A	108.13 (8)	C51A—C61A—H61A	120.00
O1B—S1B—O11B	118.73 (8)	S1B—C1B—C2B	120.05 (15)
O1B—S1B—C1B	106.63 (9)	S1B—C1B—C6B	120.34 (16)
O1B—S1B—C11B	106.91 (9)	C2B—C1B—C6B	119.51 (18)
O11B—S1B—C1B	107.67 (9)	C1B—C2B—C3B	120.5 (2)
O11B—S1B—C11B	107.47 (9)	C2B—C3B—C4B	120.1 (2)
C1B—S1B—C11B	109.20 (8)	N4B—C4B—C5B	121.4 (3)
C4A—N4A—H42A	120.00	N4B—C4B—C3B	119.6 (3)
H41A—N4A—H42A	120.00	C3B—C4B—C5B	118.9 (2)
C4A—N4A—H41A	120.00	C4B—C5B—C6B	121.0 (2)
C4X—N4X—H4X1	120.00	C1B—C6B—C5B	120.0 (2)
C4X—N4X—H4X2	120.00	S1B—C11B—C21B	119.53 (15)
H4X1—N4X—H4X2	120.00	C21B—C11B—C61B	119.31 (17)
C41A—N41A—H43A	120.00	S1B—C11B—C61B	121.05 (15)
H43A—N41A—H44A	120.00	C11B—C21B—C31B	120.49 (19)
C41A—N41A—H44A	120.00	C21B—C31B—C41B	120.6 (2)
H41B—N4B—H42B	120.00	N41B—C41B—C51B	120.5 (2)
C4B—N4B—H42B	120.00	C31B—C41B—C51B	118.88 (19)
C4B—N4B—H41B	120.00	N41B—C41B—C31B	120.7 (2)
C41B—N41B—H43B	120.00	C41B—C51B—C61B	120.2 (2)
C41B—N41B—H44B	120.00	C11B—C61B—C51B	120.52 (19)
H43B—N41B—H44B	120.00	C3B—C2B—H2B	120.00
O11'—N11'—O12'	133.5 (10)	C1B—C2B—H2B	120.00
O11'—N11'—C1F	121.8 (7)	C2B—C3B—H3B	120.00
O12'—N11'—C1F	104.7 (7)	C4B—C3B—H3B	120.00
O12F—N11F—C1F	125.6 (7)	C6B—C5B—H5B	119.00
O11F—N11F—O12F	124.3 (9)	C4B—C5B—H5B	120.00
O11F—N11F—C1F	110.1 (6)	C5B—C6B—H6B	120.00
O31F—N31F—O32F	124.4 (2)	C1B—C6B—H6B	120.00
O32F—N31F—C3F	118.24 (18)	C11B—C21B—H21B	120.00
O31F—N31F—C3F	117.33 (19)	C31B—C21B—H21B	120.00
O51F—N51F—C5F	121.1 (7)	C21B—C31B—H31B	120.00
O51'—N51F—O52'	109.4 (6)	C41B—C31B—H31B	120.00
O52F—N51F—C5F	103.0 (4)	C61B—C51B—H51B	120.00
O52'—N51F—C5F	135.4 (5)	C41B—C51B—H51B	120.00
O51'—N51F—C5F	115.0 (5)	C11B—C61B—H61B	120.00
O51F—N51F—O52F	135.9 (8)	C51B—C61B—H61B	120.00
O31C—N11C—O32C	123.96 (17)	N11'—C1F—C6F	105.3 (4)
O31C—N11C—C3C	118.02 (16)	N11F—C1F—C6F	131.2 (4)
O32C—N11C—C3C	118.01 (16)	C2F—C1F—C6F	122.8 (2)
O11C—N31C—C1C	116.3 (2)	N11'—C1F—C2F	131.8 (4)
O11C—N31C—O12C	125.8 (2)	N11F—C1F—C2F	106.1 (4)
O12C—N31C—C1C	117.9 (2)	C1F—C2F—C3F	116.9 (2)
O51C—N51C—C5C	118.15 (19)	N31F—C3F—C4F	118.23 (18)
O51C—N51C—O52C	124.4 (2)	C2F—C3F—C4F	123.3 (2)
O52C—N51C—C5C	117.4 (2)	N31F—C3F—C2F	118.5 (2)
O11D—N11D—O12D	124.08 (17)	C3F—C4F—C5F	116.01 (19)
O11D—N11D—C1D	118.57 (17)	N51F—C5F—C6F	117.7 (3)
O12D—N11D—C1D	117.35 (16)	C4F—C5F—C6F	123.5 (2)
O32D—N31D—C3D	117.2 (2)	N51F—C5F—C4F	118.7 (2)
O31D—N31D—O32D	125.0 (2)	C1F—C6F—C5F	117.6 (3)
O31D—N31D—C3D	117.86 (18)	C1F—C2F—H2F	122.00
O51D—N51D—O52D	125.4 (2)	C3F—C2F—H2F	122.00
O51D—N51D—C5D	116.9 (2)	C3F—C4F—H4F	122.00
O52D—N51D—C5D	117.8 (2)	C5F—C4F—H4F	122.00
S1A—C1A—C6X	127.0 (6)	C5F—C6F—H6F	121.00
C2A—C1A—C6A	114.2 (6)	C1F—C6F—H6F	121.00
S1A—C1A—C2X	110.2 (4)	C2C—C1C—C6C	123.08 (19)
S1A—C1A—C2A	132.7 (4)	N31C—C1C—C2C	117.67 (19)
C2X—C1A—C6X	122.8 (7)	N31C—C1C—C6C	119.1 (2)
S1A—C1A—C6A	113.1 (5)	C1C—C2C—C3C	116.67 (18)
C1A—C2A—C3A	127.5 (5)	N11C—C3C—C4C	118.63 (16)
C1A—C2X—C3X	115.2 (5)	N11C—C3C—C2C	117.75 (17)
C2A—C3A—C4A	118.1 (6)	C2C—C3C—C4C	123.44 (18)
C2X—C3X—C4X	123.4 (6)	C3C—C4C—C5C	116.95 (17)
N4A—C4A—C5A	117.0 (5)	C4C—C5C—C6C	122.29 (19)
N4A—C4A—C3A	123.0 (5)	N51C—C5C—C6C	118.22 (19)
C3A—C4A—C5A	120.0 (5)	N51C—C5C—C4C	119.38 (17)
C3X—C4X—C5X	119.9 (5)	C1C—C6C—C5C	117.6 (2)
N4X—C4X—C3X	123.6 (6)	C1C—C2C—H2C	122.00
N4X—C4X—C5X	116.6 (6)	C3C—C2C—H2C	122.00
C4A—C5A—C6A	120.6 (7)	C5C—C4C—H4C	121.00
C4X—C5X—C6X	116.2 (6)	C3C—C4C—H4C	122.00
C1A—C6A—C5A	119.6 (10)	C5C—C6C—H6C	121.00
C1A—C6X—C5X	122.6 (10)	C1C—C6C—H6C	121.00
S1A—C11A—C21A	119.15 (14)	C2D—C1D—C6D	123.37 (17)
C21A—C11A—C61A	120.05 (17)	N11D—C1D—C2D	118.66 (17)
S1A—C11A—C61A	120.79 (14)	N11D—C1D—C6D	117.95 (16)
O12E—N11E—C1E	117.2 (2)	C1D—C2D—C3D	117.14 (19)
O11E—N11E—O12E	125.0 (2)	N31D—C3D—C4D	119.01 (18)
O11E—N11E—C1E	117.8 (2)	C2D—C3D—C4D	122.50 (18)
C11A—C21A—C31A	119.96 (18)	N31D—C3D—C2D	118.5 (2)
C21A—C31A—C41A	120.87 (18)	C3D—C4D—C5D	117.37 (18)
O32E—N31E—C3E	117.32 (18)	N51D—C5D—C4D	119.52 (19)
O31E—N31E—O32E	124.5 (2)	N51D—C5D—C6D	117.56 (18)
O31E—N31E—C3E	118.2 (2)	C4D—C5D—C6D	122.91 (19)
C31A—C41A—C51A	118.35 (16)	C1D—C6D—C5D	116.67 (17)
N41A—C41A—C51A	120.82 (17)	C3D—C2D—H2D	121.00
N41A—C41A—C31A	120.82 (17)	C1D—C2D—H2D	121.00
C41A—C51A—C61A	120.65 (17)	C3D—C4D—H4D	121.00
O52E—N51E—C5E	118.28 (18)	C5D—C4D—H4D	121.00
O51E—N51E—O52E	124.29 (19)	C5D—C6D—H6D	122.00
O51E—N51E—C5E	117.44 (17)	C1D—C6D—H6D	122.00
C11A—C61A—C51A	120.11 (18)	N11E—C1E—C2E	118.68 (18)
C1A—C2A—H2A	116.00	N11E—C1E—C6E	118.33 (18)
C3A—C2A—H2A	116.00	C2E—C1E—C6E	122.98 (19)
C1A—C2X—H2X	122.00	C1E—C2E—C3E	117.25 (18)
C3X—C2X—H2X	122.00	N31E—C3E—C2E	118.68 (18)
C4A—C3A—H3A	121.00	N31E—C3E—C4E	118.98 (19)
C2A—C3A—H3A	121.00	C2E—C3E—C4E	122.33 (19)
C4X—C3X—H3X	118.00	C3E—C4E—C5E	117.58 (19)
C2X—C3X—H3X	118.00	N51E—C5E—C4E	118.71 (18)
C6A—C5A—H5A	120.00	N51E—C5E—C6E	118.52 (17)
C4A—C5A—H5A	120.00	C4E—C5E—C6E	122.76 (18)
C4X—C5X—H5X	122.00	C1E—C6E—C5E	117.04 (18)
C6X—C5X—H5X	122.00	C1E—C2E—H2E	121.00
C5A—C6A—H6A	120.00	C3E—C2E—H2E	121.00
C1A—C6A—H6A	120.00	C3E—C4E—H4E	121.00
C5X—C6X—H6X	119.00	C5E—C4E—H4E	121.00
C1A—C6X—H6X	119.00	C1E—C6E—H6E	121.00
C11A—C21A—H21A	120.00	C5E—C6E—H6E	121.00
C31A—C21A—H21A	120.00		
			
O1A—S1A—C1A—C2A	−156.7 (6)	C21A—C31A—C41A—N41A	179.94 (17)
O1A—S1A—C1A—C6A	24.6 (6)	O32E—N31E—C3E—C2E	173.00 (18)
O11A—S1A—C1A—C2A	−28.7 (7)	O31E—N31E—C3E—C2E	−7.0 (3)
O11A—S1A—C1A—C6A	152.5 (6)	O31E—N31E—C3E—C4E	171.87 (18)
C11A—S1A—C1A—C2A	88.0 (6)	O32E—N31E—C3E—C4E	−8.2 (3)
C11A—S1A—C1A—C6A	−90.8 (6)	N41A—C41A—C51A—C61A	−179.49 (17)
O1A—S1A—C11A—C21A	−31.97 (17)	C31A—C41A—C51A—C61A	−0.3 (3)
O1A—S1A—C11A—C61A	147.63 (14)	C41A—C51A—C61A—C11A	−0.7 (3)
O11A—S1A—C11A—C21A	−160.46 (15)	O52E—N51E—C5E—C6E	−8.5 (3)
O11A—S1A—C11A—C61A	19.14 (18)	O51E—N51E—C5E—C6E	171.95 (17)
C1A—S1A—C11A—C21A	82.38 (17)	O51E—N51E—C5E—C4E	−6.4 (3)
C1A—S1A—C11A—C61A	−98.03 (16)	O52E—N51E—C5E—C4E	173.22 (17)
C1B—S1B—C11B—C61B	−101.70 (16)	C2B—C1B—C6B—C5B	−1.0 (3)
O11B—S1B—C1B—C6B	−26.78 (17)	S1B—C1B—C2B—C3B	−175.63 (16)
C11B—S1B—C1B—C2B	−94.11 (16)	C6B—C1B—C2B—C3B	0.7 (3)
C11B—S1B—C1B—C6B	89.61 (17)	S1B—C1B—C6B—C5B	175.26 (16)
O1B—S1B—C11B—C21B	−32.92 (17)	C1B—C2B—C3B—C4B	0.4 (3)
O1B—S1B—C1B—C2B	21.07 (17)	C2B—C3B—C4B—C5B	−1.1 (4)
O1B—S1B—C1B—C6B	−155.22 (15)	C2B—C3B—C4B—N4B	−179.6 (2)
O11B—S1B—C1B—C2B	149.51 (15)	N4B—C4B—C5B—C6B	179.3 (2)
C1B—S1B—C11B—C21B	82.08 (17)	C3B—C4B—C5B—C6B	0.8 (4)
O1B—S1B—C11B—C61B	143.30 (15)	C4B—C5B—C6B—C1B	0.3 (3)
O11B—S1B—C11B—C21B	−161.40 (14)	C61B—C11B—C21B—C31B	−0.8 (3)
O11B—S1B—C11B—C61B	14.82 (17)	S1B—C11B—C21B—C31B	175.52 (14)
O11F—N11F—C1F—C6F	−12.2 (10)	S1B—C11B—C61B—C51B	−175.17 (14)
O12F—N11F—C1F—C2F	−10.0 (10)	C21B—C11B—C61B—C51B	1.1 (3)
O12F—N11F—C1F—C6F	171.1 (7)	C11B—C21B—C31B—C41B	−0.3 (3)
O11F—N11F—C1F—C2F	166.7 (6)	C21B—C31B—C41B—N41B	−179.27 (17)
O31F—N31F—C3F—C2F	−7.6 (3)	C21B—C31B—C41B—C51B	1.1 (3)
O31F—N31F—C3F—C4F	170.44 (19)	C31B—C41B—C51B—C61B	−0.8 (3)
O32F—N31F—C3F—C2F	173.1 (2)	N41B—C41B—C51B—C61B	179.56 (17)
O32F—N31F—C3F—C4F	−8.8 (3)	C41B—C51B—C61B—C11B	−0.3 (3)
O51F—N51F—C5F—C6F	−166.6 (8)	N11F—C1F—C6F—C5F	−180.0 (5)
O51F—N51F—C5F—C4F	15.6 (9)	C6F—C1F—C2F—C3F	−1.2 (4)
O52F—N51F—C5F—C4F	−166.0 (4)	N11F—C1F—C2F—C3F	179.7 (4)
O52F—N51F—C5F—C6F	11.8 (5)	C2F—C1F—C6F—C5F	1.3 (4)
O32C—N11C—C3C—C2C	−165.13 (18)	C1F—C2F—C3F—N31F	177.5 (2)
O31C—N11C—C3C—C4C	−170.91 (17)	C1F—C2F—C3F—C4F	−0.5 (3)
O32C—N11C—C3C—C4C	10.1 (3)	C2F—C3F—C4F—C5F	2.1 (3)
O31C—N11C—C3C—C2C	13.9 (3)	N31F—C3F—C4F—C5F	−175.89 (19)
O11C—N31C—C1C—C6C	−8.4 (3)	C3F—C4F—C5F—N51F	175.7 (2)
O11C—N31C—C1C—C2C	167.5 (2)	C3F—C4F—C5F—C6F	−2.1 (3)
O12C—N31C—C1C—C2C	−11.2 (3)	C4F—C5F—C6F—C1F	0.5 (4)
O12C—N31C—C1C—C6C	172.9 (2)	N51F—C5F—C6F—C1F	−177.3 (3)
O51C—N51C—C5C—C4C	−3.4 (3)	C2C—C1C—C6C—C5C	−0.8 (3)
O51C—N51C—C5C—C6C	172.9 (2)	C6C—C1C—C2C—C3C	1.4 (3)
O52C—N51C—C5C—C6C	−5.0 (3)	N31C—C1C—C6C—C5C	174.94 (19)
O52C—N51C—C5C—C4C	178.8 (2)	N31C—C1C—C2C—C3C	−174.40 (18)
O12D—N11D—C1D—C2D	−3.5 (2)	C1C—C2C—C3C—N11C	174.02 (16)
O11D—N11D—C1D—C2D	177.22 (16)	C1C—C2C—C3C—C4C	−1.0 (3)
O12D—N11D—C1D—C6D	175.08 (16)	C2C—C3C—C4C—C5C	0.0 (3)
O11D—N11D—C1D—C6D	−4.3 (2)	N11C—C3C—C4C—C5C	−174.94 (16)
O31D—N31D—C3D—C2D	0.8 (3)	C3C—C4C—C5C—C6C	0.7 (3)
O32D—N31D—C3D—C2D	−179.74 (17)	C3C—C4C—C5C—N51C	176.74 (17)
O31D—N31D—C3D—C4D	−177.44 (18)	C4C—C5C—C6C—C1C	−0.3 (3)
O32D—N31D—C3D—C4D	2.0 (3)	N51C—C5C—C6C—C1C	−176.43 (19)
O51D—N51D—C5D—C6D	−166.8 (2)	C6D—C1D—C2D—C3D	−1.9 (3)
O52D—N51D—C5D—C6D	12.8 (3)	N11D—C1D—C6D—C5D	−176.21 (16)
O52D—N51D—C5D—C4D	−168.6 (2)	C2D—C1D—C6D—C5D	2.3 (3)
O51D—N51D—C5D—C4D	11.8 (3)	N11D—C1D—C2D—C3D	176.53 (16)
S1A—C1A—C2A—C3A	177.5 (6)	C1D—C2D—C3D—N31D	−178.16 (16)
C2A—C1A—C6A—C5A	2.8 (14)	C1D—C2D—C3D—C4D	0.0 (3)
S1A—C1A—C6A—C5A	−178.2 (8)	N31D—C3D—C4D—C5D	179.57 (16)
C6A—C1A—C2A—C3A	−3.8 (13)	C2D—C3D—C4D—C5D	1.4 (3)
C1A—C2A—C3A—C4A	2.2 (14)	C3D—C4D—C5D—N51D	−179.61 (18)
C2A—C3A—C4A—C5A	0.8 (10)	C3D—C4D—C5D—C6D	−1.1 (3)
C2A—C3A—C4A—N4A	178.6 (7)	C4D—C5D—C6D—C1D	−0.7 (3)
C3A—C4A—C5A—C6A	−1.5 (11)	N51D—C5D—C6D—C1D	177.89 (17)
N4A—C4A—C5A—C6A	−179.4 (8)	N11E—C1E—C2E—C3E	178.05 (18)
C4A—C5A—C6A—C1A	−0.3 (14)	C6E—C1E—C2E—C3E	−1.1 (3)
S1A—C11A—C61A—C51A	−178.27 (14)	N11E—C1E—C6E—C5E	179.83 (18)
S1A—C11A—C21A—C31A	178.72 (14)	C2E—C1E—C6E—C5E	−1.1 (3)
C61A—C11A—C21A—C31A	−0.9 (3)	C1E—C2E—C3E—N31E	−179.14 (16)
C21A—C11A—C61A—C51A	1.3 (3)	C1E—C2E—C3E—C4E	2.1 (3)
O11E—N11E—C1E—C2E	−167.3 (2)	N31E—C3E—C4E—C5E	−179.66 (16)
O12E—N11E—C1E—C2E	12.5 (3)	C2E—C3E—C4E—C5E	−0.9 (3)
O12E—N11E—C1E—C6E	−168.3 (2)	C3E—C4E—C5E—N51E	176.83 (16)
O11E—N11E—C1E—C6E	11.8 (3)	C3E—C4E—C5E—C6E	−1.4 (3)
C11A—C21A—C31A—C41A	−0.2 (3)	N51E—C5E—C6E—C1E	−175.91 (16)
C21A—C31A—C41A—C51A	0.8 (3)	C4E—C5E—C6E—C1E	2.4 (3)

### Hydrogen-bond geometry (Å, °)

**Table:**

*D*—H···*A*	*D*—H	H···*A*	*D*···*A*	*D*—H···*A*
N4A—H41A···O52D^i^	0.88	2.49	3.324 (4)	160
N41A—H43A···O12D^ii^	0.88	2.46	3.248 (2)	149
N41A—H44A···O52C^iii^	0.88	2.26	3.096 (2)	158
N41B—H44B···O31C^iv^	0.88	2.58	3.028 (2)	113
N4X—H4X1···O51E^v^	0.88	2.46	3.267 (4)	152

Symmetry codes: (i) −*x*+1, −*y*+1, −*z*; (ii) −*x*+1, −*y*+1, −*z*+1; (iii) −*x*+2, −*y*+1, −*z*+1; (iv) *x*−1, *y*−1, *z*; (v) −*x*+2, −*y*+1, −*z*.
